# Transcending frontiers in prostate cancer: the role of oncometabolites on epigenetic regulation, CSCs, and tumor microenvironment to identify new therapeutic strategies

**DOI:** 10.1186/s12964-023-01462-0

**Published:** 2024-01-12

**Authors:** Giulia Ambrosini, Marco Cordani, Ali Zarrabi, Sergio Alcon-Rodriguez, Rosa M. Sainz, Guillermo Velasco, Pedro Gonzalez-Menendez, Ilaria Dando

**Affiliations:** 1 Department of Neuroscience, Biomedicine and Movement Sciences, University of Verona, 37134 Verona, Italy; 2 Department of Biochemistry and Molecular Biology, Faculty of Biology, Complutense University, 28040 Madrid, Spain; 3 Instituto de Investigaciones Sanitarias San Carlos (IdISSC), 28040 Madrid, Spain; 4 Department of Biomedical Engineering, Faculty of Engineering & Natural Sciences, Istinye University, Istanbul, 34396 Turkey; 5 Department of Research Analytics, Saveetha Dental College and Hospitals, Saveetha Institute of Medical and Technical Sciences, Saveetha University, Chennai, 600 077 India; 6 Departamento de Morfología y Biología Celular, School of Medicine, Julián Claveria 6, 33006 Oviedo, Spain; 7 Instituto Universitario de Oncología del Principado de Asturias (IUOPA), University of Oviedo, 33006 Oviedo, Spain; 8 Instituto de Investigación Sanitaria del Principado de Asturias (ISPA), Hospital Universitario Central de Asturias (HUCA), 33011 Oviedo, Spain

**Keywords:** Prostate cancer, Oncometabolites, Epigenetic alterations, Cancer stem cells (CSCs), Metabolic Enzymes, EMT

## Abstract

Prostate cancer, as one of the most prevalent malignancies in males, exhibits an approximate 5-year survival rate of 95% in advanced stages. A myriad of molecular events and mutations, including the accumulation of oncometabolites, underpin the genesis and progression of this cancer type. Despite growing research demonstrating the pivotal role of oncometabolites in supporting various cancers, including prostate cancer, the root causes of their accumulation, especially in the absence of enzymatic mutations, remain elusive. Consequently, identifying a tangible therapeutic target poses a formidable challenge. In this review, we aim to delve deeper into the implications of oncometabolite accumulation in prostate cancer. We center our focus on the consequential epigenetic alterations and impacts on cancer stem cells, with the ultimate goal of outlining novel therapeutic strategies.

**Graphical Abstract:**

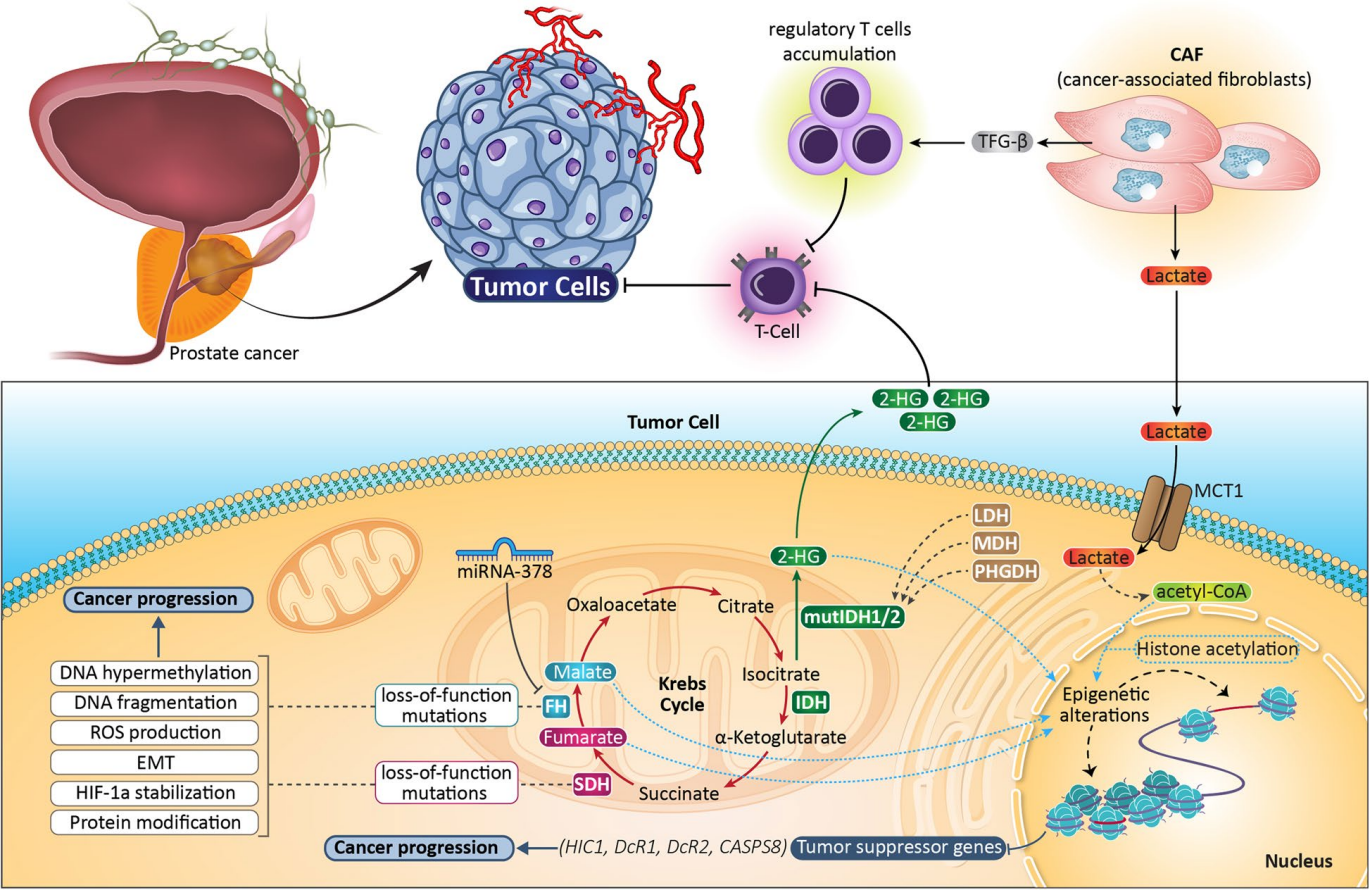

## Oncometabolites and their impact on the epigenetic landscapes of prostate cancer

### Overview of prostate cancer

The prostate is the largest accessory gland in the male reproductive system, consisting of tubulo-alveolar glands. It comprises a glandular epithelium of variable height and a stroma containing connective tissue and smooth muscle. In the adult male, the prostate is divided into four zones with different relative content in epithelial and stromal cells. Only 25% of the surface in the central zone comprises epithelial glands, while the peripheral zone contains more than 70% of epithelial tissue. The transitional and periurethral zone have few secretory tissues although they undergo extensive proliferation in older individuals [1]. Prostate cancer (PC) is a significant global health concern, predominantly affecting the older male population. It develops in the prostate, a small walnut-shaped gland in men that produces seminal fluid to nourish and transport sperm. The cancer usually grows slowly and initially remains confined to the prostate gland, where it may not cause serious harm [2]. While some types are slow-growing and may need minimal or even no treatment, other types are aggressive and can spread rapidly [3].

The most common type of PC is adenocarcinoma, making up about 99% of cases. Other subtypes include ductal adenocarcinoma, transitional cell (or urothelial) cancer, neuroendocrine cancer, and small cell prostate cancer [4]. Castration-resistant prostate cancer (CRPC) is a highly aggressive form of the disease that grows despite the level of male hormones (androgens) being suppressed to very low or undetectable levels in the body. It usually signifies that the cancer is resistant to traditional hormone therapy (also known as androgen deprivation therapy), making it significantly more challenging to manage [5–7].

The genetic underpinnings of prostate cancer are complex and multifactorial. Mutations in the BRCA1 and BRCA2 genes, often associated with breast and ovarian cancer risks, have been found to increase the likelihood of prostate cancer as well [8]. Prostate cancer pathogenesis is also influenced by the roles of specific oncogenes and epigenetic regulators, including ERG (ETS-related gene), which is a member of the ETS family of transcription factors, known for roles in development and various cancers, including prostate cancer [9] and EZH2 (Enhancer of Zeste Homolog 2), which is a part of the Polycomb group of proteins, functioning as a histone methyltransferase and playing a crucial role in gene silencing and epigenetic regulation [10]. Their interaction significantly impacts prostate cancer progression. Zoma et al. found that EZH2 methylates ERG at K362, enhancing transcription and, combined with PTEN loss, leads to invasive adenocarcinomas and CRPC progression [11]. Similarly, Xu et al. identified that EZH2 in CRPC acts as a coactivator for key transcription factors and this function is phosphorylation-dependent and necessitates an intact methyltransferase domain [12]. The TMPRSS2-ERG gene fusion event is another common genetic abnormality observed in a significant proportion of PC cases, [13]. This fusion product disrupts androgen receptor signaling and induces repressive epigenetic programs through the direct activation of EZH2 [14]. This molecular interplay between ERG and EZH2 underscores their importance in cancer progression and as targets for new therapies.

Epidemiologically, prostate cancer is the second most common cancer and the fifth leading cause of cancer-related death among men, with an estimated 1.41 million new cases and 375,000 deaths worldwide in 2019, according to the World Health Organization [4]. The prevalence is higher in developed countries, attributed largely to routine screening using prostate-specific antigen (PSA) tests [15]. Age, family history of the disease, and race are well-established risk factors for PC. Men of African descent are at a higher risk of developing prostate cancer compared to other races, and the disease is generally more aggressive in these men [16].

The progression of prostate cancer, similarly to many other types of cancer, is influenced by metabolic alterations in the tumor cells [17]. These changes allow cancer cells to adapt to their environment and to the availability of nutrients, enabling their survival and proliferation even under adverse conditions. Among the metabolic alterations seen in cancers, the accumulation of specific metabolites, referred to as 'oncometabolites', has emerged as a significant contributor to tumor progression [18]. This leads us to a deeper understanding of how prostate cancer develops and evolves, which we will explore in the next section, focusing specifically on the role of oncometabolites in tumor progression.

The understanding and management of prostate cancer have evolved significantly over the years. Today, due to enhanced screening methods and more effective treatments, survival rates for prostate cancer are generally high, particularly for localized forms of the disease [19, 20]. However, aggressive and late-stage forms, such as CRPC, present ongoing challenges that researchers are striving to address. The future of prostate cancer research involves a multidimensional approach, focusing on risk stratification, personalizing treatments, and continually developing novel therapies.

### Role of oncometabolites in tumor progression

**Fig. 1:**
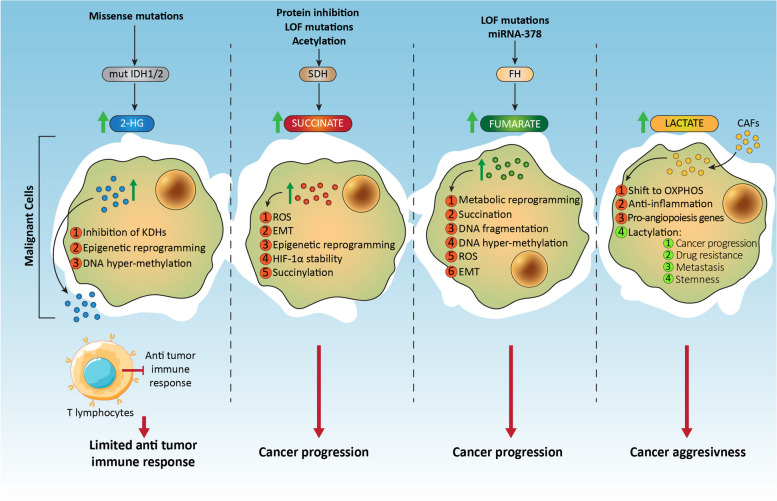
Scheme representing the role oncometabolites in prostate cancer with addition of other tissues, highlighting potential diagnostic tools

As mentioned above, among the hallmarks of cancers, metabolic alterations are emerging as one of the most intriguing and complex therapeutic targets [21, 22] due to the strict connections of the different metabolic pathways and the capacity of cancer cells to adapt their energetic requirements on nutrient availability and microenvironmental changes. Alterations of energetic metabolism are connected with several downstream pathways and regulatory mechanisms, including protein post-translational modifications, epigenetic variations, and intercellular crosstalk, corroborating the necessity to identify metabolic targets to specifically hit cancer cells, still keeping into consideration their plastic ability to change their energetic sources. In different tumor types, the accumulation of specific metabolites (called oncometabolites) has been reported, especially concerning three intermediates derived from the Krebs cycle, i.e., 2-hydroxyglutarate (2-HG), succinate, and fumarate. Historically, 2-HG has been the first identified oncometabolite; it is mainly produced by the mutated form of the enzyme isocitrate dehydrogenase (mutIDH1/2) [23] and it is structurally similar to α-ketoglutarate (α-KG), the physiological product of the wild-type IDH. Gain-of-function mutations of IDH have been identified in different tumor types, including gliomas, leukemia, chondrosarcoma, colon cancer, and prostate carcinoma [24]. Interestingly, 2-HG can be derived also from the promiscuous activity of some key metabolic enzymes, including malate dehydrogenase (MDH), lactate dehydrogenase (LDH) [25], and phosphoglycerate dehydrogenase (PHGDH) [26], and can accumulate in cancer cells in both its enantiomeric forms (i.e., D- and L-2HG). It has been reported that D-2HG is released by malignant cells and accumulated in the extracellular space, then it is taken up by T lymphocytes, limiting their capacity to mediate antitumoral immune response (Fig. 1) [27]. In addition to 2-HG, also high levels of succinate and fumarate have been shown to sustain cancer growth. For both, the cause is connected to alterations of the functionality of the relative enzymes, such as succinate dehydrogenase (SDH) and fumarate hydratase (FH), respectively, that include loss-of-function mutations, transcript degradation by miRNAs, post-translational modifications, and protein degradation. Concerning SDH, its loss-of-function mutations have been detected mainly in two rare tumors, such as paraganglioma and pheochromocytoma [24], and are generally frequent on the two subunits SDHB and SDHC, whereas only few mutations affect the other subunits (SDHA and SDHD) [28]. This enzyme is localized into mitochondria and acts in two crucial pathways of the energetic metabolism, such as the Krebs cycle and the electron transport chain, by converting succinate into fumarate, together with the reduction of flavin adenine dinucleotide (FAD) into FADH_2_. A blockage of SDH activity causes an accumulation of succinate, which then acts as an oncometabolite by activating signaling pathways, DNA hypermethylation, increase of reactive oxygen species (ROS) production, epithelial-to-mesenchymal transition (EMT), hypoxia-inducible factor (HIF)-1α stabilization, protein modification (such as succinylation) to sustain cancer progression (Fig. 1) [28]. Similarly, the accumulation of fumarate is generally caused by loss-of-function mutations of the relative enzyme FH, which converts fumarate into malate, and these mutations have been evidenced mainly in neuroblastoma and glioblastoma [24]. In addition, FH expression can be decreased by miRNA-378*. The results of the low activity of FH determine the increased level of fumarate supporting cancer spread through metabolic reprograming, post-translational modifications (succination), DNA fragmentation and hypermethylation, increase of ROS levels and EMT (Fig. 1) [28].

Otto Warburg won the Nobel Prize in 1931 for his discovery of the increased glycolytic rate of cancer cells accompanied by high levels of lactate production, even in presence of oxygen. Recently, due to its abundance in tumor specimens and to its capacity to influence cancer cell metabolism, lactate has also been categorized as an oncometabolite. Indeed, environmental lactate not only acts as a metabolic by-product, but it can be uploaded by lung, prostate, and pancreatic cancer cells in order to support metabolic pathways, including fatty acid metabolism [29–31]. In uveal melanoma, it has been demonstrated that lactate supplementation to in vitro cultured cells increases the mRNA expression of factors involved in mitochondria biogenesis and activity, suggesting a metabolic shift towards an oxidative metabolism [32]. In addition, in the same paper, the authors showed that the expression of monocarboxylate transporter (MCT), such as MCT1 and MCT4, are increased by lactate, along with a boost in oxidative phosphorylation (OXPHOS) activity, supporting a quiescent state and a reduced proliferation. However, other data are necessary to further corroborate this mechanism. A crucial role of lactate accumulation in cancer cells has been demonstrated regarding its ability to induce expression of anti-inflammatory and proangiogenic genes, together with the modification of histones through a process called “lactylation”, where a lactyl-CoA is added to lysine residues present in the tails of histones [33]. This epigenetic alteration results in cancer progression, drug resistance [34], metastasis and stemness induction [35, 36], metabolic alterations by affecting enzymes involved in the tricarboxylic acid cycle (TCA), and carbohydrate, amino acid, fatty acid, and nucleotide metabolism [35], hence opening the way to the generation of novel therapeutic strategies against lysine lactylation, not only regarding histones (Fig. 1) [36]. Finally, it is worth noting that other metabolic intermadiates or biochemical molecules may accumulate in cancer cells [37], thus emerging as other possible oncometabolites, hence maintaing open the inclusion in this class of pro-cancer molecules.

### Role of oncometabolites in modulating the epigenetic landscape of cancer cells

Concerning the epigenetic alterations associated with oncometabolites, it has been shown that D-2HG accumulation, an event that is coupled with α-KG depletion, inhibits histone lysine demethylases (KDMs), giving rise to histone hypermethylation, epigenetic programming, and acquisition of additional mutations [27]. Furthermore, since 2-HG is structurally similar to α-KG, it competitively inhibits some α-KG-dependent dioxygenases, including the ten eleven translocation enzymes (TETs) and the Jumonji family of histone lysine demethylases, giving rise to DNA and histone hypermethylation [38, 39]. Some evidence about the capacity of succinate to regulate the epigenetic pattern of cancer cells have also been reported. Indeed, it has been shown that the acetylation of SDHA causes its deactivation, which in turn leads to activation of H3K4me3 activation, accompanied by change in tumor-specific gene expression [40]. In addition, other authors showed epigenetic inactivation of tumor suppressor genes, specifically *HIC1, DcR1, DcR2* and *CASPS8*, due to succinate accumulation in SDH-mutated head and neck paragangliomas [41]. Concerning fumarate, instead, it has been reported a connection between it and chromatin remodeling factor lymphoid-specific helicase (LSH) in patients with nasopharyngeal carcinoma (NPC). Indeed, the authors showed that the subsequent chromatin modification has a key role in the determination of epithelial–mesenchymal transition, by conferring potent metastatic potential to cancer cells [42]. Interestingly, it has been recently demonstrated that also lactate accumulation influences the epigenetic pattern of cancer cells, in particular prostate cancer. Indeed, it has been shown that the inhibition of both the lactate transporter MCT1 and the key enzyme at the basis of lipid biosynthesis, i.e., the ATP citrate lyase (ACLY), strongly reduce the intracellular levels of acetyl-CoA, bringing the authors to the suggestion that lactate produced by cancer-associated fibroblasts (CAFs) represents the main source of acetyl-CoA in lipid-reprogrammed prostate cancer cells. Since lipid-derived Acetyl-CoA is one of the major sources of carbons for histone acetylation, the authors also showed that exogenous lactate induces increased levels of specific histones acetylation (in particular H3K9 and H3K27) that correlate with an open state of the chromatin, suggesting an active gene transcription. These interesting results are further supported by the evidence that lactate derived from CAFs enters inside cancer cells and activates this epigenetic process, highlighting that the interplay between CAFs and cancer cells is crucial and that CAFs act as key players in the dysregulation of metabolite accumulation [29].

### Impact of oncometabolites on prostate cancer progression and prognosis

Due to the impact of oncometabolites in cancers, different studies have been focused on their role in prostate cancer (Table 1). Starting from one of the first studies on this topic, it has been demonstrated that missense mutations on codon 132 in *IDH1* gene are present in different tumor types diverse than glioblastoma multiforme or gliomas, including prostate carcinomas [43], opening the way to the investigation of the role of 2-HG also in this tumor type. Indeed, thereafter, many papers showed the effect of *IDH* mutation, and thus 2-HG production, on prostate cancer cells. Among these, it has been demonstrated that the TET family of α-KG dioxygenases catalyzes the oxidation of 5-methylcytosine to 5-hydroxymethylcytosine correlating with DNA demethylation. Interestingly, TET2 is considered as a tumor suppressor gene and its enzymatic activity is inhibited in IDH-mutated tumors by the accumulation of 2-HG; this is further connected with the low levels of 5-methylcytosine oxidation that sustains cancer development, including prostate [44]. Another interesting evidence has been reported about the link of *IDH* mutation on codon 132 or of 2-HG high levels and the higher cell invasion capacity of prostate cancer cells negative for the androgen receptor (AR); indeed, the authors showed that when AR is expressed it reverses the pro-tumorigenic effect of 2-HG via transforming growth factor (TGF)β1 and circRNA-51217 [45].

**Table 1:** Publications on PubMed regarding oncometabolites and prostate cancer

Argument	Number of reviews	Year of publication
Prostate cancer stem cells	695	#59 in 2021 #54 in 2022 #19 in 2023 (‘til now)
Prostate cancer and oncometabolites	2	2019
Prostate cancer and epigenetic	705	#69 in 2021 #75 in 2022 #33 in 2023 (‘til now)
Prostate cancer and tumor microenvironment	902	#137 in 2021 #118 in 2022 #52 in 2023 (‘til now)

Concerning succinate, a lower number of studies are present in the literature. In one of these, the authors reported that after the therapeutic inhibition of AR, there is a metabolic shift in the regulation of energy metabolism, highlighted by lower levels of SDH activity, correlating with succinate accumulation. Hence in this scenario, a therapeutic approach could also have a negative loop effect by causing an accumulation of a molecule that could work oppositely respect to the beneficial effect of the drug [46]. Moreover, also the anti-oxidant enzyme superoxide dismutase 2 (SOD2) has been shown to be indirectly involved also in the sustainment of succinate accumulation through a decreased activity of SDH and, intriguingly, an enhanced expression of Glut1 and glucose uptake in prostate cancer models [47], however these evidence need further validations in other biological systems. In this frame, CAFs and succinate accumulation has been reported play a key role in the context of prostate cancer. Indeed, CAFs establish a metabolic connection with prostate cancer cells, through lactate shuttle and thus contributing to cancer aggressiveness. Specifically, it has been shown that lactate uptake by cancer cells alters the NAD^+^/NADH ratio, followed by sirtuin (SIRT)1-dependent peroxisome proliferator-activated receptor gamma coactivator (PGC)-1α activation and subsequent increase of mitochondrial mass and activity. This scenario is then reflected on the deregulation of Krebs cycle, accumulation of oncometabolites, downregulation of complexes II-III, and accumulation of ROS. In addition, the authors also showed that CAFs stimulated prostate cancer cells to invade and that CAFs conditioned media significantly enhanced malate, citrate, fumarate and succinate levels (not 2-HG) [48].

Three other types of metabolic alterations have been demonstrated in prostate cancer cells: i) increased levels of urea cycle intermediates, including arginine-succinate, arginine, and fumarate in prostate cancer cells in comparison to benign controls [49]; ii) overproduction of γ-aminobutyric acid (GABA) suggesting that the GABA shunt, particularly glutamate decarboxylase (GAD)65, may represents a molecular target in the treatment of castrate-resistant prostate cancer [50]; iii) the accumulation of a derivate of the amino acid glycine, i.e. N-methyl-glycine, also called sarcosine, which has been shown to accumulate in prostate neoplastic cells acting as an apoptotic inhibitor [51] and as an epigenetic modifier [52], thus representing a potential biomarker for the diagnosis of this cancer in the early stage [53]. Finally, in a pilot study it has been shown that, by analyzing the urine of 101 patients with prostate cancer, the concentration of sarcosine, together with ethanolamine, kynurenine, b-alanine, and isoleucine, is significantly different respect 52 control individuals, representing new potential markers for cancer detection and prognosis [54]. This aligns with the findings that activation of the PTEN-PI3K-mTORC1 pathway in prostate cancer leads to metabolic alterations in polyamine synthesis and decarboxylated S-adenosylmethionine (dcSAM) production, further underscoring the potential of metabolic markers in cancer detection and evaluation [37].

## Oncometabolites and epigenetic regulation in cancer stem cells (CSCs) and EMT

### Cancer stem cells in the context of prostate cancer

Despite prostate cancer usually grows slowly without spreading outside the gland, there are also more aggressive forms, in which cancer cells rapidly invade the surrounding tissues and can additionally spread to other organs. Some studies have therefore focused on the investigation of this particular and delicate clinical condition, until the discovery in 2005 by Collins and colleagues of prostate cancer stem cells (PCSCs) [55] insights into the understanding and nature of these cells have followed since then. Cancer stem cells are extremely rare within the tumor, indeed they represent only the 0.1–1% of the bulk of the malignant mass and possess specific peculiarities. Among these, they show self-renew capacity and are able to differentiate into non-CSC progeny; they have high tumor-initiation capacity and display a strong resistance to chemo- and radio-therapy. In fact, standard therapies generally kill differentiated cancer cells, whereas cancer stem cells survive giving rise to metastasis. All these elements determine a worse clinical outcome for cancer pathology. As a matter of fact, it is now commonplace to think that the portion of patients with prostate cancer who are in a metastatic stage and who, above all, do not respond to hormonal therapy (considered a gold standard) are distinguished precisely by the presence of cancer stem cells [56]. This makes them one of the main targets in the development of new possible therapies for the treatment of patients to whom it is not possible to apply the classic treatment strategies nowadays. The cure and complete healing of prostate cancer still remains an incomplete success, but one that can be resolved by unmasking the intrinsic properties of PCSCs [56].

There are several types of cells in the prostate, each of which can transform and become cancerous; it therefore agrees that one of the main characteristics of this type of tumor is the relevant heterogeneity, which manifests itself in a range of different stadial degrees named Gleason grade and metastatic states [57]. In order to identify and analyze prostate cancer stem cells, specific markers have been established over the years that allow their isolation; among them, CD44, CD133, aldehyde dehydrogenase (ALDH), and androgen receptors are noteworthy but not the only ones. The origin of PCSCs is not yet well clear, although emerging data suggest the idea that they derive from prostate stem cells that undergo epigenetic modifications due to the presence of singular molecules released in the tumor microenvironment (TME) [58].

### Role of EMT in prostate cancer progression and metastasis

One of the main traits for cancer cells is their attitude to undergo epithelial-to-mesenchymal transition, a well-recognized pathway implicated in the formation of CSCs. During this physiological event, cells decrease the expression of specific epithelial proteins and instead increase the expression of other mesenchymal specific proteins. This leads cells to lose tight and adherent junctions and detach from the basement membrane, consequently disrupting cell polarity; then, they migrate throughout the bloodstream and extravasate, colonizing secondary organs [59]. To do this, cells must undergo the opposite mechanism, which is the mesenchymal-to-epithelial transition (MET). Over last years, many papers demonstrated that also prostate cancer cells possess the propensity to undergo EMT program, endowing tumor with invasive traits that facilitate metastasis and variation in therapeutic responses [60]. In particular, it is emerging that androgen-independent metastatic phenotype, advanced prostate cancer and resistant immuno-phenotype patients are associated with the presence of PCSCs [61].

During the normal process of prostate formation, which occurs at embryonic level, it is normal to witness a dynamic plasticity between EMT and MET that allows the formation of the endodermal and mesodermal structures [62]. However, when some of the crucial signaling pathways that regulate these processes are significantly altered, patients undergo prostate tumorigenesis. First of all, the Wnt pathway, that generally affects cell survival, and in particular the Wnt family member 3 (Wnt3) seems to be involved in the increased expression of some typical stem markers like CD133 and CD44 [63–65]. The Hedgehog signaling pathway orchestrates cell renewal and survival, and its abnormal regulation has been found in prostate cancer cells and in PCSCs [66, 67], correlating with poor survival because of high grade of metastasis. Interestingly, also Notch pathway is regulated in prostate cancer; indeed, recent data show that it alters the correct signaling of both phosphoinositide 3-kinase (PI3K)/Akt pathway and AR pathway, which coordinate carcinogenesis [68], EMT, metastasis and cancer progression in this tumor type [69–72]. A final pathway that is altered in prostate cancer is nuclear factor kB (NF-kB) signaling pathway, which is correlated with a complex clinical case since it appears to have an important role in the regulation of progression, in the formation of metastases, and also in resistance to therapies [73, 74]. Further studies need to be performed to deeply investigate this topic which appears to be as crucial and equally important as those previously mentioned.

Since important pathways are significantly altered during EMT, one of the approaches that is emerging recently is the search for specific markers involved in the aforementioned pathways in order to use them as targets not only in patient screening, to better understand in which tumor stage are classified, but also as therapeutic targets to improve life expectancy even for those patients who are resistant to therapies and/or show relapses [75]. Moreover, in last years, in-depth studies on animal models have also been performed to further understand the effects that EMT and CSCs can have on prostate cancer [76], identifying eligible markers for possible future patient-specific treatments, in a historical moment in which the precision medicine seems to be the new frontier in the fight against cancer.

### Influence of oncometabolites and epigenetic changes on CSCs and EMT

As previously stated, the complexity of tumors often lies in the high heterogeneity that characterizes it and which, among others, is due to the coexistence of cancer cells and cancer stem cells. In addition to this, the sophisticated molecular and metabolic plasticity that CSCs undergo makes the clinical profile more complex. The mechanisms underlying this fluidity can be traced back to epigenetic alterations that provide a reversible and rapid switch in gene expression, in response to developmental and microenvironmental signals, as well as to hypoxic conditions [77–79]. These events trigger the alteration of specific oncogenes; these genes can be functional and active during physiological conditions, producing normal amounts of metabolites. Their upregulation, as a consequence of epigenetic modifications, can result in an overabundance of key metabolites, known as oncometabolites, and lead to tumorigenesis. In fact, in addition to acting as signal molecules in TME, they also act on signaling pathways which in turn orchestrate both the expression of stem traits and their intracellular abundance in a metabolic loop. This metaboloepigenetic rewiring is called “metabostemness” [80]. In detail, epigenetic modifications lead to mutations on enzymes involved in metabolic pathways (among which the best noted are SDH, FH, and IDH) which in turn cause the accumulation of oncometabolites (as mentioned before, succinate, fumarate, and 2-hydroxyglutarate) that stimulates alterations in DNA and histone modifications [28]. Each of the above oncometabolites regulates certain signaling pathways, including those regulating stemness and the EMT program. For example, succinate and fumarate inhibit TETs, important methylcytosine dioxygenases that not only demethylate DNA but also suppress specific microRNAs that usually inhibit EMT program [81]; this interference causes a constant activation of epithelial-to-mesenchymal transition and an increased expression of *Zeb1*, which has been identified as a potential risk factor for recurrence and poor prognosis in several types of cancers [82, 83]. In the case of 2-hydroxyglutarate instead, it seems to induce an abnormal activity of telomerase reverse transcriptase (TERT), which in turn upregulates the transcription of important genes involved in stemness and tumorigenicity, including CD117, Oct4, and Sox2 [84, 85].

This complexity is further elucidated in a recent study by Giafaglione et al., which demonstrates that prostate epithelial cells, specifically basal and luminal types, possess unique metabolomes and nutrient patterns, fundamentally impacting their differentiation and response to treatment. These findings highlight the significant role of metabolic signaling in the regulation of prostate cancer progression, especially in the context of treatment resistance and lineage identity [86].

In recent years there have also been studies on possible link between some mutations found in patients with therapy-resistant prostate cancer, and a higher level of expression of genes involved in stemness, EMT and invasiveness [61]. These data are alarming but, at the same time, they also leave a glimmer of hope for the identification of markers and/or therapeutic targets that can help in the fight against this type of cancer (Table 2).

**Table 2:** Role of oncometabolite in cancer cells related to epigenetic landscape

Role of oncometabolites in cancer cells (epigenetic landscape)	Reference
2-hydroxyglutarate (2-HG)	
Limiting T lymphocytes capacity to mediate antitumoral immune response	[27, 38]
Inhibition of histone lysine demethylases (KDMs)	
Inhibition of ten eleven translocation enzymes (TETs) and the Jumonji family of histone lysine demethylases	[39]
Succinate	
DNA hypermethylation	[28]
Increase of reactive oxygen species (ROS) production	
Induction of epithelial-to-mesenchymal transition (EMT)	
Hypoxia-inducible factor (HIF)-1a stabilization	
Protein modification (such as succinylation)	
Inhibition of H3K4me3 activation, accompanied by change in tumor-specific gene expression	[40]
Inactivation of tumor suppressor genes (*HIC1*, *DcR1*, *DcR2* and *CASPS8*)	[41]
Fumarate	
Metabolic reprograming	[28]
Post-translational modifications (succination)	
DNA fragmentation and hypermethylation	
Increase of reactive oxygen species (ROS) levels	
Induction of epithelial-to-mesenchymal transition (EMT)	
Connection with chromatin remodeling factor lymphoid-specific helicase (LSH)	[42]
Lactate	
Inducing expression of anti-inflammatory and proangiogenic genes	[33]
Modification of histones (lactylation)	
Drug resistance	[34]
Induction of metastasis and stemness	[35, 36]
Supporting metabolic alterations	[35]
Increasing levels of specific histones acetylation (in particular H3K9 and H3K27)	[29]

## The impact of oncometabolites on the tumor microenvironment in prostate cancer

### Elucidating the composition and metabolic function of the prostate cancer tumor microenvironment

The stroma of the human prostate is a dense connective tissue rich in smooth muscular cells with fibroblast, blood vessels, and nerves. There is no adipose tissue in the stroma, though the periprostatic adipose tissue of the prostate plays a fundamental role in the biology of the gland. In the mouse, the fibromuscular stroma is less prominent, and the epithelial tissue occupies most of the gland area [87].

Stromal cells are responsible for the growth and homeostasis of the epithelia. Also, they are responsible for extracellular matrix synthesis and turnover. The importance of the stroma in the normal gland functioning is obvious. Growth factors travel around and are stacked into the fibrous component of the extracellular matrix, the communication between stroma and epithelia is also relevant in the aging process of the gland. Aging changes matrix components and is characterized by increased pro-inflammatory cytokines that can contribute to age-associated pathologies such as benign prostate hyperplasia, prostatitis, and even prostate carcinoma [88]. In fact, cancer progression involves signals and interaction with other cell types different from tumor cells found in the neighboring tissue that embrace the tumor. In the prostate, epithelial cancerous tissues are surrounded by cells that produce factors that communicate and even regulate tumor cells [89].

The importance of TME in PC has made the number of studies focused on the metabolic crosstalk between cancer cells and the TME rapidly increase in the last few years. The TME maintains the metabolic requirements of PC cells in different ways: (1) by the production of many cytokines; (2) by secreting lactate; (3) by ensuring intracellular alkalinization of tumoral cells through bicarbonate secretion via carbonic anhydrase IX (CAIX) [90].

The unique metabolism of PC entails that the relevant oncometabolites in the TME differ from other carcinomas. In normal prostate, glycolysis is favored because of the inhibition of aconitase in the TCA cycle by zinc accumulation [91]. On the other hand, in the first steps of prostate carcinogenesis, tumor cells become citrate-dependent, increasing OXPHOS and lipogenesis. In the CRPC, choline, amino acid, and glycolytic metabolism are promoted more than OXPHOS [92]. Thus, the different metabolic requirements along PC progression alter the TME, being the mitochondria at the center of the crosstalk.

In PC, most published studies are based on the interaction of tumor cells with CAFs to promote cancer progression and aggressiveness. In healthy prostate stroma, fibroblasts play a fundamental role in the maintenance of the extracellular matrix (ECM), but acquire an activated state with tumor progression that resembles its role during the repair response in injured tissues. CAFs increase the production of EMC components and secrete growth factors and cytokines [93]. In the prostate stroma, they increase proliferation and begin to express α smooth muscle actin (α-SMA), a classical marker of myofibroblast [94]. CAFs are found in premalignant prostatic intraepithelial neoplasia (PIN), but they are significantly increased along with tumor progression [95, 96]. They express high levels of EMC proteins and show high mobility and a high proliferation rate similar to fibroblast during wound healing, but the main difference is that CAFs remain in the stroma of the TME [97]. This situation maintains oncogenic signals, including soluble factors produced by themselves or by modifying the availability of oncogenic molecules bound to the ECM components. The origin of CAFs remains unclear, and some propose that they derive from the activation of resident fibroblast [98, 99], from recruited bone marrow progenitors [100], or the transdifferentiation from endothelial [101, 102] or epithelial to mesenchymal [103].

CAFs show a secretory phenotype that enhances the proliferation and invasion of prostate cancer cells in vitro*.* However, though in vivo, CAFs have been positive or negative for tumor growth, depending on the study. The role of CAFs in grafting-initiated prostate epithelium has been shown [104, 105]. The role of CAFs is to stimulate the cancer cells. They contribute to a permissive microenvironment that favors angiogenesis, cell migration, matrix stiffening, and in turn invasive phenotypes [95, 96]. CAF-enriched microenvironment directly affects EMC integrity, promoting fibrosis, interstitial pressure, hypoxia and impairing the delivery of antitumor drugs.

On the other hand, proteases from CAFs degrade ECM components and free growth factors and cytokines that contribute to proliferation of cancer cells. Lastly, CAFs, through this microenvironment modulation, could be relevant in the immune population and reactivity surrounding the tumor cells, however their role in immunosuppression in prostate cancer is not yet clarified.

The second component in the prostate cancer microenvironment is tumor vasculature. Like many other solid tumors, the prostate has aberrant vasculature showing a lack of pericyte coverage, vascular leakiness, and aberrant morphology of vessels [106]. In normal prostate, vascular endothelial growth factor (VEGF) expression is restricted to stromal cells. However, in carcinoma, VEGF expression increases in tumor cells. Prostate cancer favors angiogenesis, and tumor-associated vasculature secretes an array of paracrine effectors that can contribute to the growth of several types of solid tumors [107]. Experimentally, it was demonstrated that human umbilical vein endothelial cells enhance the invasion of prostate cancer cells in vitro [108]. The integration of vascular niche in the prostate is affected by androgen deprivation therapy. Androgens withdrawal promotes the loss of vascular integrity, pointing to the possibility that the vascular component of the prostate microenvironment can be an important component of treatment-induced therapy resistance since some experiments have shown a transient loss of vascular integrity that is recovered after a time of androgen deprivation [109]

The changes in vascularization make the environment hypoxic and acidic [110]. In general, it was considered that tumor cells adapt their metabolism favoring glycolysis to have a selective advantage in a non-physiological atmosphere, being glucose considered as oncometabolite. However, glucose is not frequently viewed as oncometabolite in PC, except for metastases [111]. In fact, glucose deprivation promotes androgen signaling and increases resistance to radiation treatment [112, 113]. Lactate and fatty acids predominate over glucose as energetic fuel in PC cells.

Both CAFs and the vascular component of the TME live together with the immune cells of the prostate stroma. Inflammation is a cancer-promoting process in the prostate. Chronic infection and inflammation cause cancer in the prostate [114]. Also, the progression of prostate tumors is followed by the increment of immune cells in the stroma, mostly dendritic cells and macrophages. Macrophages have a complex role in cancer, but in the prostate, tumor-associated macrophages (TAM) are frequently correlated with poorer outcomes [115]. Macrophages interact with fibroblasts in normal tissues when tissue repair is necessary. TFG-β and other cytokines mediate this interaction. CAFs produce high levels of TFG-β that correlate with lower survival in prostate cancer patients. TFG-β also promotes the accumulation of regulatory T cells in the tumor prostate. Thus, the reciprocal relation between stromal components contributes to progression and creates an immunosuppressive microenvironment [89]. Histology evidence reflects that more than 80% of prostate human samples studied have immune infiltration, and this chronic inflammation contributes to prostate cancer development [116]. The composition of immune infiltrate varies with disease stage and patient age, but mostly, CD3^+^ T cells, CD4^+^ T cells, CD20^+^ B cells, and macrophages are found [117]. Other frequent cells that appear in the TME of the prostate are natural killers (NK), mast cells and neutrophils that, in addition to macrophages, constitute the most relevant immune components of the stroma of the prostate tumor. Immune cell function is finely regulated by nutrient availability, and targeting metabolic activity is a recurrent strategy for malignant cells to proliferate.

One of the major immunosuppressive components in the prostatic TME is the dedifferentiated myeloid-derived suppressor cells (MDSCs). These cells, coming from myeloid cells in the bone marrow, are highly heterogeneous and can also differentiate into endothelial cells, fibroblasts, tumor-associated macrophages (TAMs) [118], or osteoclasts [119]. Consequently, MDSCs play a critical role in forming the pre-metastatic niche. According to their morphology, MDSCs are subdivided into two major subsets: Monocytic (M-MDSCs) and granulocytic (G-MDSCs). Despite their heterogeneity, MDSCs metabolically share a Warburg effect exhibition, which limits glucose availability for immune cells and the production of inhibitory cytokines by increasing fatty acid oxidation (FAO). In addition, the promotion of the catabolism of some amino acids, such as arginine, tryptophan, and cysteine, by MDSCs impedes activation of T cells [120]. In PC, several studies have already described the presence of MDSCs to support tumor growth [121]. Interestingly, enzalutamide, an AR antagonist used in metastatic PC, promotes the immunosuppressive activity of MDSCs by reducing mitochondrial metabolism and increasing glycolysis, VEGF and Arg1 expression in a CRPC model [122]. Therefore, the resistance to AR antagonism can be explained by MDSC metabolic function.

The biophysical properties of the stroma, like the cell communication between all the components of the stroma of the tumor prostate, play a critical role not only in the progression of the disease but also in the resistance of the tumor to radiation or cytotoxic therapy. This mechanism relies on the role of ECM synthesized by cellular components and the communication between these components and cancer cells. There is a growing interest in the communication mechanism between cancer cells and stromal cells. The role of extracellular vesicles (EVs), membrane-enclosed particles secreted by several cells, including cancer epithelial, immune, or non-immune cells, is interesting. EVs can carry cytoplasmic proteins, lipids, nucleic acids, or metabolites that affect cells in the same vicinity or far distance. They also have a role in modulating tumor immune response and have many functions, including oncogenic signals, angiogenesis, or tumor-evading signals. They are critical mediators by which tumors regulate their microenvironment and, in return, by which stromal cells modulate tumors [123, 124]. For example, exosomes from MDSCs ease CRPC progression [125]. On the other hand, Xu and colleagues recently described how exosomes from PC cells containing interleukin-8 (IL-8) alter the metabolism of stromal CD8^+^ T cells, switching from OXPHOS to thermogenesis [126].

### Role of oncometabolites in shaping the tumor microenvironment and cancer metastasis

From the classical point of view of the Warburg Effect, lactate has been considered a waste product from glycolytic tumor cells which acidifies the TME. Lactic acidosis in the TME promotes metastasis, angiogenesis, and immunosuppression [127]. However, lactate can also be catabolized to pyruvate, fueling the TCA cycle and stimulating different anabolic signals [128]. In 2008, Sonveaux et al. showed that lactate uptake is preferred in OXPHOS-dependent cancer cells and is involved in cellular processes such as redox homeostasis [129]. In 2017, Hui et al. proposed lactate as the primary carbon source in the TCA cycle [130].

**Fig. 2:**
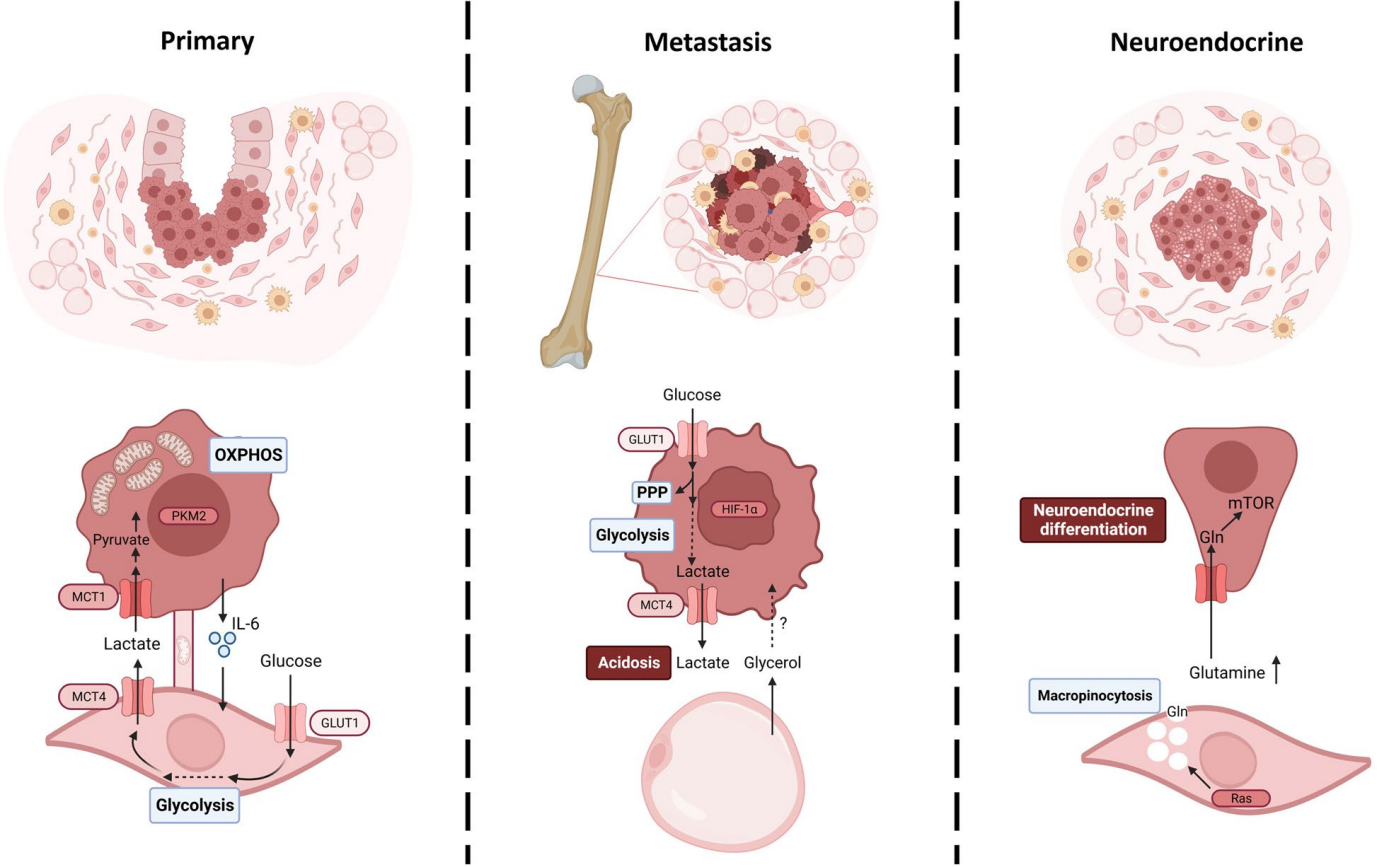
Oncometabolites in the tumor microenvironment

The lactate uptake, known as Reverse Warburg Effect, is particularly relevant in the prostatic TME and different from other tumors [131]. PC cells modify the metabolism of CAFs by Interleukin-6 (IL-6) secretion [132]. Epithelial tumor cells downregulate the tumor suppressor p62 in fibroblasts. Consequently, there is a reduction in the mTORC1 activity and c-Myc expression that causes a release of ROS and IL-6 and the activation of fibroblasts [133]. CAFs enhance glucose transporter GLUT1 and lactate exporter MCT4 levels, becoming glycolytic and secreting lactate [134, 135]. CAFs also activate Pyruvate kinase M2 (PKM2) in PC cells. This protein is translocated into the nucleus, losing its metabolic function and switching the metabolism to OXPHOS [134, 136]. Simultaneously, PC cells reduce GLUT1 levels and increase the levels of the lactate importer MCT1, consuming the lactate secreted by CAFs (Fig. 2) [134]. In 2019, Ippolito et al. studied the role of lactate coming from CAFs into tumor cells more in-depth. Lactate impacts the NAD^+^/NADH ratio, activating the mitochondrial regulator PGC-1α via SIRT1. Consequently, there is an increase in mitochondrial biomass and activity, an accumulation of oncometabolites from the TCA cycle, and more superoxide generation. Interestingly, along with lactate uptake, tumor cells established cellular bridges with CAFs to hijack functional mitochondria [48]. Although still not completely defined, this metabolic phenotype seems characteristic of primary tumors. The metastatic and CRPC forms secrete lactate via MCT4 and present reduced levels of MCT1 [137, 138].

The lactic acidification in the TME also impacts immunomodulation, particularly associated with TAMs. Acidosis alters interleukin-4-related macrophage activation (M2-immunosuppressive phenotype), promoting tumor growth. In addition, there is a negative correlation between macrophage infiltration and MCT4 levels (Fig. 2) [139]. Recently, Chaudagar et al. found an inverse correlation between glycolytic activity and antitumor TAM phagocytosis in metastastic CRPC (mCRPC) patients [140].

Besides lactate production, CAFs feed cancer cells through ketone bodies, molecules necessary to ensure lipid synthesis under glucose deprivation [141, 142]. Lipids are metabolites highly present in the tumor stroma, particularly in bone metastases [143]. While CAFs are the most abundant cells of the TME of primary tumors, adipose tissue is the majority in the bone marrow, where PC metastasis is more frequent. The high metabolic activity of the adipocytes promotes the proliferation and aggressiveness of cancer cells in bone metastasis, being proposed as responsible for the metabolic disbalance from OXPHOS to glycolysis [144]. In vitro, PC cells in the presence of adipocytes are more glycolytic by increasing HIF1α levels. However, physical interaction is not necessary, but the specific factors involved in PC progression are not elucidated yet [145]. Cutruzzolà et al. suggest adipocyte-secreted glycerol can feed cancer cells by entering the glycolytic pathway [144]. On the other hand, Whitburn and colleagues described that bone stromal cells upregulate glucose-6-phosphate dehydrogenase activity and, consequently, the pentose phosphate pathway (PPP) by IL-6 secretion (Fig. 2) [146].

Another oncometabolite related to lipid metabolism is choline, which is part of the phospholipid phosphatidylcholine. Choline is highly abundant in PC cells [147], and the TME influences choline metabolism [148]. However, it has not been elucidated which component regulates choline levels. Furthermore, PC cells secrete extracellular lipid vesicles enriched in Caveolin-1, phosphatidylcholine, sphingolipids, and cardiolipins [149]. Therefore, the tumor stroma regulates choline metabolism in PC cells, but choline secretion can also impact the TME. On the other hand, cholesterol is also highly concentrated in PC cells [150], and CAFs upregulate its synthesis and the steroid biosynthesis pathway [151], possibly by proinflammatory cytokines and chemokines, not via oncometabolite signaling.

Some amino acids are essential in shaping the TME in several cancers. Glutamine, the most abundant amino acid, plays a significant role in tumor cells but also the TME. Epigenetic regulation of Ras activity in prostatic CAFs metabolically links the stroma and the tumoral epithelia. The fibroblastic Ras-driven macropinocytosis triggers an increase in glutamine production. The glutamine excess is employed by tumor cells to promote tumor progression, neuroendocrine differentiation, and resistance to androgen deprivation therapy (ADT) (Fig. 2) [152]. Moreover, arginine, a semi-essential amino acid, is particularly relevant in immune cells [153]. Arginine metabolism is the metabolic bridge between malignant PC cells and TAM, increasing tumor aggressiveness [154]. Arginase (ARG) 1 and ARG2, enzymes responsible for catabolizing arginine into ornithine, activate immunosuppressive pathways in the hormone-refractory PC [155]. In addition, arginine is also in control of maintaining the unresponsiveness of tumor-infiltrating cytotoxic lymphocytes. In tumor cells, inhibition of ARG and nitric oxide synthase (converts arginine into nitric oxide) promotes lymphocyte responsiveness by reducing tyrosine nitration [156]. In mice, extracellular arginine is taken by MDSCs via upregulation of cationic amino acid transporter 2 (Cat2/Slc7a2), reducing its availability for immune cells [157]. In patients, it was also confirmed that high levels of Arg1 in a G-MDSC population inhibit the proliferation and cytotoxic activity of CD8 + T cells [158].

The function of famous oncometabolites like 2-hydroxyglutarate, fumarate, or succinate has not been extensively described for the PC microenvironment. Succinate from stromal cells can have a remarkable goal in oncogenic potential since PC cells consume it in an acidic environment, having an anaplerotic effect and enhancing the aggressive potential [159, 160]. Succinate can be secreted from CAFs through exosomes. CAFs-derived exosomes include lactate, acetate, amino acids, TCA cycle metabolites like succinate, and lipids. Furthermore, exosome secretion is pH dependent, being favored under an acidic environment. Unlike the lactate effect alone, CAFs-derived exosomes inhibit the electron transport chain, increasing glutamine carboxylation for biosynthesis in PC cells [161].

### Influence of oncometabolites in CSC phenotype

Even if the impact of oncometabolites in the crosstalk between stromal cells and PC cells has been reasonably assessed, less clear is which are the oncometabolites responsible for maintaining the metabolic properties of CSCs. CSCs are generally characterized by a high glycolytic rate and low OXPHOS [162], but their metabolism is also tissue-dependent [163]. Thus, unlike PC cells, glucose can be a critical metabolite for stemness. The CSC cell line 3-AB OS, derived from the human osteosarcoma cell line MG63, is sensitive to glucose deprivation [164]. However, MG63 cells are more susceptible to glutamine deprivation [165]. Recently, it was described in the prostatic PC-3 cell line that cell density shifts metabolism from glycolysis towards OXPHOS and induces a CSC-like phenotype. However, it seems that glycolysis and not OXPHOS is critical for CSC phenotype since dichloroacetate, a pyruvate dehydrogenase inhibitor, blocks the acquisition of CSC properties [166]. Nevertheless, this study was performed in vitro with only one cell line, requiring more studies to extract consistent conclusions.

Among the candidates to regulate CSCs in PC, kynurenine, a metabolite from tryptophan metabolism, is more abundant than lactate in the PC microenvironment and can have immunosuppressive properties, promoting malignancy [167, 168]. Kynurenine has been involved in self-renewal, maintenance, and differentiation in embryonic stem cells (ESC) and induced pluripotent stem cells (iPSCs) [168, 169]. Therefore, this metabolite can be a major candidate for regulating CSCs in the prostate.

## Therapeutic implications and future perspectives

### Current Strategies targeting metabolic dysregulation, oncometabolites, and epigenetic modulation in prostate cancer

#### Biomarkers

Recent technical breakthroughs in analytical chemistry allow us to identify low-abundance metabolites in different biological matrixes and metabolic alterations along key pathways [170]. Since oncometabolites are characteristic of tumor development and progression, identifying their differential levels in diverse biological samples compared to healthy individuals could be an interesting approach as novel prostate cancer biomarkers. Here we sum up the oncometabolites differently identified in preclinical models and in urine, seminal fluid, and biopsies of PC patients.

Urine, as part of the urogenital system and an easy-to-get sample, could be a feasible matrix to look for patient biomarkers. Succinate is detected and less abundant in PC patients' urine (*n* = 32) compared to healthy controls (*n* = 32). The study's authors explained this as an indication of the disruption of the TCA cycle in tumor cells [171]. Interestingly, they also found hydroxyglutarate downregulated in the urine of PC patients. Kyneurine, another oncometabolite [172, 173], was found to have significantly higher median values in the urine of PC patients (*n* = 101) than in controls (*n* = 52) [54].

Due to the significant contribution of the prostate to the seminal fluid composition, it is reasonable to search for potential biomarkers within it. Citrate emerges as a widely differentially produced metabolite in seminal fluid. Although citrate is not an oncometabolite, it is a critical factor in prostate metabolism. In normal prostate, it is accumulated in epithelial cells due to zinc-induced TCA cycle blockade at the m-aconitase level. Citrate, along with zinc, is secreted to seminal fluid. However, metabolic reprogramming during PC initiation drops zinc and citrate levels, unblocking the TCA cycle [174]. Thus, citrate has lower levels in PC patients' seminal fluid than in control groups across different studies (PC *n* = 4, control *n* = 22 [175]; PC *n* = 28, control *n* = 33 [176]; PC *n* = 52, control *n* = 26 [177]; and PC *n* = 3, control *n* = 4 [178]).

Molecular and metabolic profiling of cancer biopsies is a novel opportunity to identify new features of solid tumors. Fumarate increased in patient cancer samples compared to adjacent benign tissue (*n* = 13) [49]. Furthermore, this oncometabolite positively correlated with gene expression of oncogenic HIF1ɑ and NF-kB pathways [49]. Tessem et al., 2016, tried to shed light on the metabolic profiling of solid tumors and find biomarkers despite different and complex mixtures of tissues within the sample. By selecting samples with a similar proportion of tumor and stroma across biopsies and groups, they could identify differentially elevated levels of succinate and reduced levels of citrate between PC patients (*n* = 95) and healthy individuals (*n* = 34) [179]. Finally, another oncometabolite linked to in situ tumor progression is lactate. Despite its low concentration, using transrectal ultrasound-guided biopsies, lactate was identified, and PC samples contained differentially higher levels than benign tissue (*n* = 82). Interestingly, they could reach an ability to detect this increase in lactate with as little as 5% of tumor content in the biopsy, making it a promising tool for PC detection [180].

Together with the advances in detection methods of metabolites, the field of EVs is also growing thanks to better and refined methods of isolation, characterization, and their new roles discovered in many hallmarks of cancer. Thus, PC patient-derived EVs isolation and characterization is being used as a biomarker search, as reviewed in [181]. Indeed, Clos-Garcia et al., 2018, isolated EVs from the urine of PC patients and then performed a metabolomic analysis of their cargo. Succinate could be detected in all samples (PC *n* = 31 and BPH *n* = 14), but with a fold-change of 1.211, the *p*-value of the comparison PC vs BPH was 0.11. Citrate and isocitrate were also identified, and interestingly they were significantly downregulated in EVs from PC [182]. Other oncometabolites detected in EVs are lactate and fumarate, identified in high concentrations in patient-derived CAFs, which are incorporated by PC cells in vitro to fuel their metabolic demands [161]. Furthermore, succinate has been identified in EVs derived from PC cells in vitro, with significantly higher levels than non-tumoral counterparts [183]. These findings make the characterization and search of specific (onco)metabolites in EVs of PC patients a stimulating research field.

#### Therapeutic agents

Once identified as potential biomarkers, the mechanical effects of oncometabolites on tumor initiation and progression also make them attractive therapeutic targets. Not only the classic oncometabolites and their downstream effects but also the different pathways related to the wide metabolic dysregulation that occurs along PC can offer an opportunity to understand more of the weaknesses of this tumor, trying to find new efficient candidate treatments.

Metabolic rewiring of glucose, lipids, and amino acids, among others, is a hallmark of PC. Early stages of PC, contrary to most tumors, are characterized by oxidative phosphorylation-based metabolism rather than aerobic glycolysis, while advanced CRPC stages rely on glycolysis [174, 184]. De novo lipogenesis and fatty acid oxidation (FAO) are early PC events, also correlating with disease progression [185, 186]. Glutamine, the most abundant amino acid in plasma, plays a crucial role in PC metabolism since it can be used as a substitute for glucose in TCA cycle, and as a building block for nucleotide synthesis and fatty acid production [186, 187]. Thus, targeting this metabolic dysregulation at different levels could become a practical therapeutic approach.

Focusing on using TCA cycle derivatives and oxidative phosphorylation, the mitochondrial electron transport chain could be an effective target in PC cells. There are several compounds showing effectiveness in preclinical studies. MSDC-0160 is an inhibitor of the mitochondrial pyruvate carrier (MPC), which breaks the link between cytosolic and mitochondrial metabolism. This drug reduces proliferation by preventing cell cycle progression and disrupts the TCA cycle in AR-driven PC models (cell lines and xenografts) [188]. Electron transport chain (ETC) complex-I inhibitors rotenone, its derivative deguelin, and IACS-010759 inhibit proliferation and induce apoptosis in PTEN-null models of PC [189, 190]. Metformin, which inhibits ETC complex-I and is the most common anti-diabetic drug, is being studied in 33 clinical trials as an anti-PC agent, and 8 of them have available results so far. Some remarkable results are the decline of PSA after combination of metformin with bicalutamide after 8 weeks (42.1% bicalutamide + metformin *vs* 11.1% only bicalutamide) in PC patients with overweight (ClinicalTrial.gov ID NCT02614859), and with castration after 7 months (placebo PSA 58.2 ng/mL vs metformin PSA 8.36 ng/mL, ClinicalTrial.gov ID NCT01620593, [191]), and its detection in the prostatic tissue after consumption for 12 weeks, but without changes in tumor progression markers (ClinicalTrials.gov ID NCT01433913, [192]). However, other studies finished without concluding beneficial results, making it necessary to study refinement mechanisms further to explode the potentiality of metformin or identify potential patient candidates that could be benefited from metformin treatment. In vitro, some of the mechanisms of action described is to effectively induce p53-dependent apoptosis in PC cells when combined with 2-deoxyglucose, inhibiting glycolysis and mitochondrial respiration together [193, 194].

As an energy sensor, 5’AMP-activated kinase (AMPK) exerts many actions widely regarded as tumorigenic suppressors. Selective AMPK-activator MT 63–78 inhibits proliferation and induces apoptosis in PC cells by disrupting lipogenesis [195]. Moreover, due to the dependence on liponeogenesis of PC cells, fatty acid synthase (FASN) inhibitors are showing promising results. C75-dependent FASN blockade reduces the proliferation of LNCaP cells and tumor growth in vivo [196]. Another orally available, reversible, selective FASN inhibitor, TVB-3166, inhibits anchorage-dependent cell growth and induces apoptosis in PC cell lines and patient-derived xenografts through different downstream mechanisms [197]. Finally, FASN inhibitor TV-2640 is currently in phase I clinical trial (ClinicalTrial.gov ID NCT05743621). The study aims to determine the effects of this drug together with AR-inhibitor enzalutamide in mCRPC. Fatty acid oxidation is another attractive target. The commercially available drug ranolazine is employed for heart angina, but decreased tumor growth in a xenograft PC model [198] and sensitized to antiandrogen treatment in vitro and in a xenograft [199] when administered orally. This drug inhibits carnitine palmitoyltransferase (CPT1) and, thus, lipid oxidation.

Moreover, Telaglenastat (CB-839) is a promising orally available small molecule that inhibits glutaminase 1 (GLS1). It induces autophagy and has anti-tumor activity in multiple preclinical tumor models, including PC, alone or in combination with other treatments [200]. Telaglenastat is currently in a Phase II clinical trial, testing the effectiveness of its combination with talazoparib, a poly-ADP ribose polymerase (PARP) inhibitor, in mCRPC patients (ClinicalTrial.gov ID NCT04824937).

Regarding classic oncometabolites and metabolic rewiring, succinate is accumulated due to antiandrogen treatments, representing another therapeutic approach. Saxena et al., 2021, showed that AR transcriptionally regulates SDH subunits SDHA and SDHB. Antiandrogen therapy suppresses SDH, causing succinate accumulation. Succinate triggers a response of survival and adaption to treatments through a complex cascade that activates AR co-chaperone p-Hsp27. This way, combining antiandrogens with the p-Hsp27 inhibitor Ivermectin could be a potential improvement against antiandrogen resistance, as shown in preclinical models of LNCaP xenografts [46].

The interplay between TME, epigenetics, and cancer cells is also essential. Androgen-deprivation therapy can produce epigenetic changes in CAFs. Hypermethylation of RASAL3 promoter, a tumor suppressor that inhibits Ras function, induces glutamine secretion by CAFs. This glutamine is incorporated via facilitated transport by cancer cells, which is then derived to TCA cycle. Selectively targeting glutamine uptake by L-γ-Glutamyl-p-nitroanilide (GPNA), an inhibitor of glutamine transporter SLC1A5, showed a reduction of tumor proliferation in a preclinical 3D model and tumor growth suppression in a xenograft when combined with castration of mice [152]. Another study by Chaudagar et al., 2023, explores the potential advance inhibiting PI3K-dependent lactate production in cancer cells. As previously described, lactate secreted by PC cells is incorporated by TAMs in the TME, inducing the epigenetic change of histone lactylation and further reducing TAMs phagocytic and immunosuppressive effect on the tumor cells. Targeting PI3K pathway with Copanlisib along with ADT reduces lactate secretion and, thus, tumor growth repression helped by immunosuppression of activated TAMs in a PTEN/p53-deficient murine PC model [140].

Cells with low or absent AR expression and IDH1 R132H mutation are more likely to accumulate R-2HG than those with intact AR signaling [45]. The oncometabolite R-2HG induces PC AR^−^ cells invasion through TGFβ1/p-Smad2/3 signaling in vitro. In fact, AR^−^ cells carrying IDH1 R132H mutation metastasized more than their wild-type counterparts in a xenograft model. Since R-2HG is responsible for this phenomenon, the authors of this work propose the study of Tibsovo (ivosidenib). FDA has already approved this IDH1 inhibitor for patients with refractory acute myeloid leukemia (AML) who have IDH1 mutation(s) as a candidate drug to be tested in combination with bipolar antiandrogen therapy [45]. Targeting IDH1 mutations in PC can also have a beneficial impact on epigenetic imbalance. An epigenomic study of mCRPC patients identified a subset of CpG Methylator Phenotype (CMP), this is, with a high methylation profile, which harbored exclusive mutations in IDH1 among others (TET2 or BRAF) [201]. Altogether, IDH1 could be a therapeutic target in the small subset of IDH-mutant PCa patients by potentially improving the response to ADT reducing R-2HG and epigenetic imbalance.

### Future directions: exploiting the Interplay of oncometabolites, epigenetic changes and CSCs for therapeutic purposes

Understanding the complex interplay of oncometabolites, epigenetic changes, and CSCs has provided groundbreaking insights into the mechanistic pathways and gene regulatory networks underlying the development and progression of prostate cancer.

The recognition that oncometabolites play a central role in reprogramming the cancer epigenome has spurred the concept of 'metabostemness'. This phenomenon underscores the reciprocal interaction between cancer metabolism and stemness, where aberrant metabolites can reshape the epigenetic landscape to bolster stemness traits and promote a more malignant phenotype [80].

Oncometabolites like succinate, fumarate, and 2-hydroxyglutarate, arising due to mutations in metabolic enzymes like SDH, FH, and IDH, have been linked to changes in DNA and histone modifications, further leading to the activation of EMT and enhancement of cancer stemness properties [28]. One of the potential therapeutic strategies could be to develop small molecule inhibitors or therapeutic agents targeting these mutated enzymes, thereby curbing the production of oncometabolites and subsequently counteracting cancer progression.

Such a strategy has been demonstrated in the case of cholangiocarcinoma, where IDH1 mutations resulted in the production of the oncometabolite 2-hydroxyglutarate, leading to epigenetic alterations and changes in the expression of key genes involved in cell differentiation and survival [202]. Based on these findings, small-molecule inhibitors of the IDH1 mutated enzyme are currently under investigation in preclinical and clinical phases as promising treatments for IDH1-mutated intrahepatic cholangiocarcinomas [203, 204]. Interestingly, this approach is mirrored in a recent phase 3 trial for IDH-mutant grade 2 gliomas, where the inhibitor vorasidenib demonstrated significant improvements in progression-free survival and delay in the need for further anticancer intervention [205]. A similar approach could be contemplated for prostate cancer, with the development of specific inhibitors targeting the metabolic enzymes associated with the production of detrimental oncometabolites.

Moreover, the identification of specific markers associated with CSCs and EMT could provide potential therapeutic targets [206]. Notably, therapies could be designed to target these markers, thus providing a means of eliminating the CSC population, attenuating EMT, and curbing metastasis [207]. Such therapies could also potentially reverse therapy resistance, considering the role of CSCs in this phenomenon.

The recent revelations about the association between some mutations in therapy-resistant prostate cancer patients, higher levels of gene expression involved in stemness, EMT, and invasiveness [83, 208], also provide an opportunity to discover new targets for therapy. There is a need for the development of a precision medicine approach to tackle prostate cancer, wherein these potential therapeutic targets can be exploited to devise patient-specific treatments.

Furthermore, as CSCs have shown remarkable adaptability to various microenvironmental signals, it is equally important to study the TME and its influence on the CSC phenotype and epigenetic landscape. These studies could provide clues on how to make the tumor environment less conducive for the growth and proliferation of CSCs.

In conclusion, future research needs to intensify efforts on studying the complex interplay between oncometabolites, epigenetic changes, and CSCs. The key lies in exploiting these intricate relationships to identify potential therapeutic targets and develop effective treatment strategies for not only prostate cancer, but also other types of cancer where similar mechanisms are involved. The dawn of precision medicine offers a new hope in the fight against cancer, and the focus should be on exploring innovative and targeted approaches to capitalize on the wealth of knowledge we have gained so far.

## Conclusions

Our comprehensive review discusses the interplay between oncometabolites, epigenetic changes, and CSCs in prostate cancer and offers several key insights with significant implications for understanding and managing this prevalent disease. Firstly, we have elucidate the concept of 'metabostemness', a novel paradigm that underscores how metabolic dysregulation in cancer can perpetuate stem cell-like traits, leading to more aggressive and therapy-resistant tumor phenotypes.. The discovery that oncometabolites, such as succinate, fumarate, and 2-hydroxyglutarate, can induce significant epigenetic modifications offers a novel view on cancer pathogenesis. These insights highlight the potential of targeting metabolic pathways as a novel therapeutic strategy, as occur in cholangiocarcinoma [209, 210] and may be translated to prostate cancer treatment. Additionally, we discuss the critical role of specific genetic and epigenetic determinants in prostate cancer progression. We provide evidence that therapy-resistant prostate cancer patients often harbor certain mutations, and exhibit higher levels of genes associated with stemness, EMT, and invasiveness. This finding not only advances our molecular understanding of the disease but could be leveraged to discover new targets for therapy, paving the way for a more precise approach to prostate cancer treatment.

Another crucial aspect of our review is the emphasis on the TME and its influence on CSCs. Our findings suggest that modulating the TME could be key to inhibiting CSC growth and proliferation, thereby impeding cancer progression and metastasis.

In essence, this review contributes to the existing literature by integrating various molecular aspects of prostate cancer into a coherent framework. It underscores the importance of a multi-faceted approach encompassing genetic, metabolic, and epigenetic factors in developing more effective therapeutic strategies. As we move towards an era of precision medicine, the insights gained from this review not only enhance our understanding of prostate cancer but also highlight critical research areas that warrant further exploration. This work establishes a cornerstone for subsequent investigations, bolstering optimism for the enhanced treatment and care of prostate cancer patients globally.

## Abbreviations

2-HG: 2-Hydroxyglutarate
α-KG: α-Ketoglutarate
α-SMH: α-Smooth muscle actin
ACLY: ATP citrate lyase
ADT: Androgen deprivation therapy
ALDH: Aldehyde dehydrogenase
AMPK: 5’-AMP-activated kinase
AML: Acute myeloid leukemia
AR: Androgen Receptor
ARG: Arginase
BPH: Bening prostatic hyperplasia
CAF: Cancer-asssociated fibroblast
CAIX: Carbonic anhydrase IX
Cat2: Cationic amino acid transporter 2
CMP: CpG Methylator Phenotype
CPT1: Carnitine palmitoyltransferase
CSC: Cancer stem cell
circRNA: Circular RNA
CRPC: Castration-resistant prostate cancer
ECM: Extracellular matrix
EMT: Epithelial-to-mesenchymal transition
ERG: ETS-related gene
ESC: Embryonic stem cell
ETC: Electron transport chain
EV: Extracellular vesicle
FAD: Flavin adenine dinucleotide
FAO: Fatty acid oxidation
FASN: Fatty acid synthase
FH: Fumarate hydratase
GABA: γ-Aminobutyric acid
GAD: Glutamic acid decarboxylase
GLS-1: Glutaminase 1
G-MDSC: Granulocytic MDSC
GPNA: L-γ-Glutamyl-p-nitroanilide
HIF: Hypoxia inducible factor
IDH: Isocitrate dehydrogenase
IL-6: Interleukin-6
IL-8: Interleukin-8
iPSC: Induced pluripotent stem cell
KDM: Histone lysine demethylase
LDH: Lactate dehydrogenase
LSH: Lymphoid-specific helicase
mCRPC: Metastatic castration-resistant prostate cancer
MCT: Monocarboxylate transporter
MDH: Malate dehydrogenase
MDSC: Myeloid-Derived Suppressor Cell
MET: Mesenchymal-to-epithelial transition
M-MDSC: Monocytic MDSC
MPC: Mitochondrial pyruvate carrier
MutIDH1/2: Mutated form of the enzyme isocitrate dehydrogenase
NF-kB: Nuclear factor kB
NK: Natural killer
NPC: Nasopharyngeal carcinoma
OXPHOS: Oxidative phosphorylation
PARP: Poly-ADP ribose polymerase
PC: Prostate cancer
PCSC: Prostate cancer stem cell
PGC1α: Peroxisome proliferator-activated receptor γ co-activator 1 α
PHGDH: Phosphoglycerate dehydrogenase
PI3K: Phosphoinositide 3-kinase
PIN: Prostatic intraepithelial neoplasia
PKM2: Pyruvate kinase M2
PPP: Pentose phosphate pathway
PSA: Prostate specific antigen
ROS: Reactive oxygen species
SIRT: Sirtuin
SOD: Superoxide dismutase
SDH: Succinate dehydrogenase
TAM: Tumor-associated macrophages
TCA: Tricarboxylic acid cycle
TERT: Telomerase reverse transcriptase
TET: Ten eleven translocaion-enzyme
TGF: Transforming growth factor
TME: Tumor microenvironment
VEGF: Vascular endothelial growth factor
Wnt3: Wnt family member 3
